# FIKElectricity: A Electricity Consumption Dataset from Three Restaurant Kitchens in Portugal

**DOI:** 10.1038/s41597-023-02698-8

**Published:** 2023-11-08

**Authors:** Lucas Pereira, Vitor Aguiar, Fábio Vasconcelos, Ricardo Martins, Toni Garcês, Hugo Morais

**Affiliations:** 1 Instituto Superior Técnico, Lisbon, 1049-001 Portugal; 2 Interactive Technologies Institute, LARSyS, Lisbon, 1900-500 Portugal; 3 Agência Regional para o Desenvolvimento da Investigação, Tecnologia e Inovação, Funchal, 9020-105 Portugal; 4 Empresa de Eletricidade da Madeira, Funchal, 1960-190 Portugal; 5 Instituto de Engenharia de Sistemas e Computadores: Investigação e Desenvolvimento em Lisboa, Lisbon, 1049-000 Portugal

**Keywords:** Energy grids and networks, Electrical and electronic engineering

## Abstract

Industrial Kitchens are very energy-intensive businesses, consuming between five and seven times more energy per square meter than other commercial spaces like office spaces and retail stores. Still, very little research has been carried out on improving the energy efficiency of this industry. This paper presents the FIKElectricity dataset, a collection of electricity data from three Portuguese restaurant kitchens during their daily operation. The duration of the datasets spans three to four weeks in each industrial kitchen and comprises aggregated and individual appliance consumption, collected at 1 Hz and $\frac{1}{5}$ Hz, respectively. The public release of FIKElectricity is expected to draw more attention from the research community to this overlooked industrial sector. The data collection and post-processing methods are thoroughly described in this paper, as well as the dataset organization. Examples showing the dataset’s quality and instructions for its reuse are also provided.

## Background & Summary

In an era where the energy sector is responsible for the majority of Green House Gases (GHG) emissions^1^, and the effects of climate change are becoming widely visible^2^, improving the sustainability of high-consuming businesses has become critical^3,4^. Industrial Kitchens (IKs) stand out as one of the most energy-intensive sectors, consuming approximately five to seven times more per square meter than other commercial establishments such as office buildings and retail stores^5^. More importantly, the energy demand of such spaces is poised to surge in the foreseeable future as a consequence of the expansion in the food and beverage services market that is projected to reach $4,651.03 billion by 2027, with a Compound Annual Growth Rate (CAGR) of 5.4%^6^.

Still, despite the size of the catering industry, very little research exists towards improving the energy efficiency of its underlying processes^7^. A few noticeable exceptions are a couple of works that either attempted to develop standardized energy efficiency benchmarks^8–11^, or aimed at devising strategies to reduce and shape energy demand^12,13^. Finally, some authors have also looked at the possibility of including Renewable Energy Sources (RESs) in the operation of IKs^14–17^.

The Future Industrial Kitchen (FIK) and the Exploring the Human-Water-Energy Nexus in Industrial Kitchens (nexIK) research projects were placed in the Portuguese restaurant kitchen sector. The main goal of these two projects was to understand the interactions between electricity and water consumption and waste generation in such spaces. To this end, in the FIK project, electricity, water, and waste monitoring technology were deployed in three restaurants from Funchal, Portugal, for consecutive periods between three and four weeks^18^. The same electricity monitoring platform was later deployed in another restaurant in Lisbon in the scope of the nexIK project, which is still ongoing^19^.

This data descriptor presents the data collected through the real-time monitoring of electricity consumption in the three IKs where the deployment was initialy conducted. More precisely, aggregated (i.e., total) and disaggregated (i.e., per appliance) measurements from the three IKs in Funchal. The aggregated consumption data is available in one-second intervals, whereas the individual appliance data is available at roughly one sample every five seconds. The water consumption and waste generation measurements are publicly available in previously published data descriptors^20,21^.

To the best of our knowledge, FIKElectricity is the only publicly available dataset with electricity demand from industrial kitchens. In fact, a search on data.world (see https://data.world/search) and openei.org (see https://openei.org/) for the keywords “kitchen electricity” and “restaurant electricity” did not reveal any relevant results that suggested the existence of such datasets. This greatly contrasts the residential and office sectors, where several datasets can be found. For example, the AMPds^22^ from Canada, the REFIT^23^ from the U.K., SustDataED2^24^ from Portugal, the BLOND in Germany^25^, and the MOB dataset from the USA^26^.

Ultimately, it is expected that the public release of this dataset can draw more attention from the research community to this overlooked industrial sector. For example, this dataset can be used to validate and improve existing energy efficiency benchmarking methodologies, where despite the research efforts, it is still impossible to find a widely accepted normalization factor for energy consumption^11^. Furthermore, it can also be used to develop and evaluate methodologies to understand how appliances are used in IKs, and with that, help identify opportunities to promote a more efficient use of such devices^13,27^.

Surprisingly, despite being considered in the literature as one of the pillars for energy efficiency in IKs^28^, research in integrating Distributed Energy Resources (DERs) is also very scarce. In this context, the FIKElectricity dataset offers an opportunity to explore not only the benefits of the direct integration of DERs like Solar Photovoltaic (PV) and Battery Energy Storage Systems (BESSs) but also develop and assess new methods to enable the participation of IKs in Demand Response (DR) programs^19,29^. Moreover, a critical aspect of RES integration is the ability to predict electricity demand with different forecasting horizons. As such, this dataset provides a unique opportunity to develop and evaluate forecasting algorithms in the context of IKs, e.g.^30^.

From a monitoring perspective, it is not expected that every IK will have the resources (physical and monetary) to install circuit-level meters. In this regard, this dataset can also be used to explore if and how Non-Intrusive Load Monitoring (NILM)^31,32^, which identifies the electricity consumption of individual appliances taking only aggregated demand measurements, can be applied in IKs. Furthermore, if combined with the FIKWater dataset, there is also an exciting opportunity to explore how water and electricity consumption data can be combined to disaggregate wet appliances (e.g., dish washer and glasswasher).

This paper thoroughly describes how the FIKElectricity was collected, including detailed information on post-processing and organizing the data to form the dataset. This paper also analyzes the data quality and provides instructions for its reuse.

## Methods

### Data collection setup: aggregated consumption

The aggregated consumption data was collected using the Fluke 438-II Power Quality Analyzer & Motor Analyzer (see https://www.fluke.com/en-us/product/electrical-testing/power-quality/438-ii). This device enables the monitoring of several power-related variables, including current, voltage, active power, reactive power, power factor, voltage, and current imbalance, among others.

The collected data is stored locally on an SD Card in csv format, creating one file daily. For this particular project, the sampling rate was set to 1 Hz. At the end of each deployment, the files were downloaded and uploaded to a shared folder in the cloud.

### Data collection setup: appliances consumption

The appliance-level data was performed using the eGauge Pro circuit-level smart-meter (see https://www.egauge.net/commercial-energy-monitor/). The eGauge Pro can measure the power of up to 30 individual circuits using sensors called Current Transformers (CTs). The JD-SCT-010-0075 (75 A/0.39”) model, which has a 1% accuracy, was used in this case. The eGauge Pro enables the monitoring of current, voltage, active power, reactive power, apparent power, and power factor, among others. The data was retrieved from the eGauge Pro using the Modbus RTU protocol (see https://www.rtautomation.com/technologies/modbus-rtu/) at the rate of approximately $\frac{1}{5}$ Hz (i.e., new samples every five seconds).

**Fig. 1:**
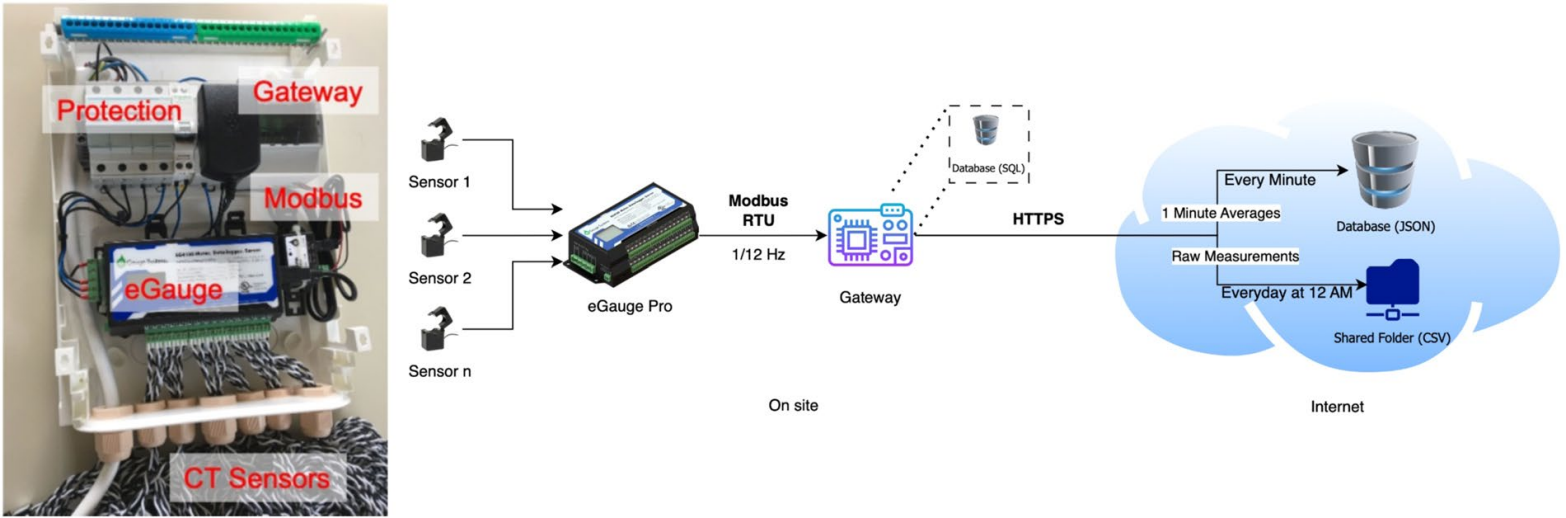
Overview of the data collection setup for individual appliance consumption. Monitoring hardware (left), and main components of the data collection setup (right).

Figure 1 illustrates the main components of the individual appliances’ data collection setup. The CTs are installed in the main breaker box (one per single-phase appliance and three per three-phase appliance). The Raspberry Pi3 gateway is connected to the eGauge Pro via USB using an RS485 (Modbus RTU) to USB converter (see https://store.egauge.net/USB_485). The gateway sequentially requests the last measurement for each circuit, taking around two seconds to scan all 30 circuits. A new scan is requested every five seconds, and the timestamp observed at the request time is shared among all the collected records. This ensures that all the records from a single scan have the same timestamp. Since the meter has a limited number of registers (60), measuring all the quantities from the meter was impossible. Instead, the following measurements were requested from the meter, in RMS values: current (*I*) in each phase, voltage (*V*) in each phase, and active power (*P*) in each phase. From there, the remaining quantities, i.e., apparent power (*S*), reactive power (*Q*), and power factor (*PF*), were calculated using Eqs. (1–3), respectively, for each monitored appliance (*a*) at timestep *t*.

1 $${S}_{phase}^{(a)}(t)={V}_{phase}(t)\times {I}_{phase}^{(a)}(t)$$

2 $${Q}_{phase}^{\left(a\right)}\left(t\right)=\sqrt{{S}_{phase}^{(a)2}\left(t\right)-{P}_{phase}^{(a)2}\left(t\right)}$$

3 $$P{F}_{phase}^{(a)}(t)=\frac{{P}_{phase}^{(a)}(t)}{{S}_{phase}^{(a)}(t)}$$

At this point, it’s important to note that because the eGauge meter has a restricted number of registers, obtaining power factor values for every phase was not feasible. Consequently, the reactive power and power factor had to be derived by using the apparent and active power, which inherently returns only positive values. In other words, all the *Q* vectors are assumed to be inductive and share the same direction. As a result, the total absolute value of *Q* (as defined in Eq. 5) will be the sum of the absolute values of *Q* in each individual phase (as described in Eq. 2).

The collected and calculated samples are then stored in the gateway in a relational database. The stored measurements are uploaded to an online database server every minute to provide real-time access to the consumption data^33^. Finally, every day at 12 AM, a csv file with the daily readings for each appliance is uploaded to a shared folder. Once all the records are uploaded, the local database is cleaned to keep its footprint as small as possible.

### Data post-processing

Since both monitoring setups generated one csv file per day, the first step was to merge all the daily files into single csv files.

The aggregated consumption data is made available as it was collected from the devices, i.e., no post-processing was applied. Regarding the individual appliance consumption data, three post-processing steps were required: (1) cleaning, (2) calibration, and (3) total calculation.

In the data cleaning step, all the records that contained negative values in the monitored quantities (current, voltage, and active power) were dropped. Negative values occur due to technical limitations of the monitoring hardware that are out of our control. Still, these represent only a tiny fraction of the collected data (0.041% IK 1, 0.271% IK 2, and 0.0029% IK 3).

The second step was calibrating the current measurements due to the reported accuracy of the used CTs. More precisely, since the used CTs have a reported accuracy of ±1% (corresponding to 0.75 A), all the current measurements below that value and the corresponding active power were set to zero.

Finally, the third step was to calculate the total values for each of the quantities, which was done using Eqs. (4–7).

4 $${P}_{total}^{(a)}(t)=\left\{\begin{array}{lc}{P}_{phase}^{(a)}(t) & {\rm{,\; if}}\;{\rm{single}}\;{\rm{phase}}\;{\rm{appliance}}\\ {P}_{phase\_1}^{(a)}(t)+{P}_{phase\_2}^{(a)}(t)+{P}_{phase\_3}^{(a)}(t) & {\rm{,\; if}}\;{\rm{three}}\;{\rm{phase}}\;{\rm{appliance}}\end{array}\right.$$

5 $${Q}_{total}^{(a)}(t)=\left\{\begin{array}{lc}{Q}_{phase}^{(a)}(t) & {\rm{,\; if}}\;{\rm{single}}\;{\rm{phase}}\;{\rm{appliance}}\\ {Q}_{phase\_1}^{(a)}(t)+{Q}_{phase\_2}^{(a)}(t)+{Q}_{phase\_3}^{(a)}(t) & {\rm{,\; if}}\;{\rm{three}}\;{\rm{phase}}\;{\rm{appliance}}\end{array}\right.$$

6 $${S}_{total}^{(a)}(t)=\left\{\begin{array}{lc}{S}_{phase}^{(a)}(t) & {\rm{,\; if}}\;{\rm{single}}\;{\rm{phase}}\;{\rm{appliance}}\\ \sqrt{{P}_{total}^{(a)}{(t)}^{2}+{Q}_{total}^{(a)}{(t)}^{2}} & {\rm{,\; if}}\;{\rm{three}}\;{\rm{phase}}\;{\rm{appliance}}\end{array}\right.$$

7 $$P{F}_{total}^{(a)}(t)=\frac{{P}_{total}^{(a)}(t)}{{S}_{total}^{(a)}(t)}$$

### Deployments

**Fig. 2:**
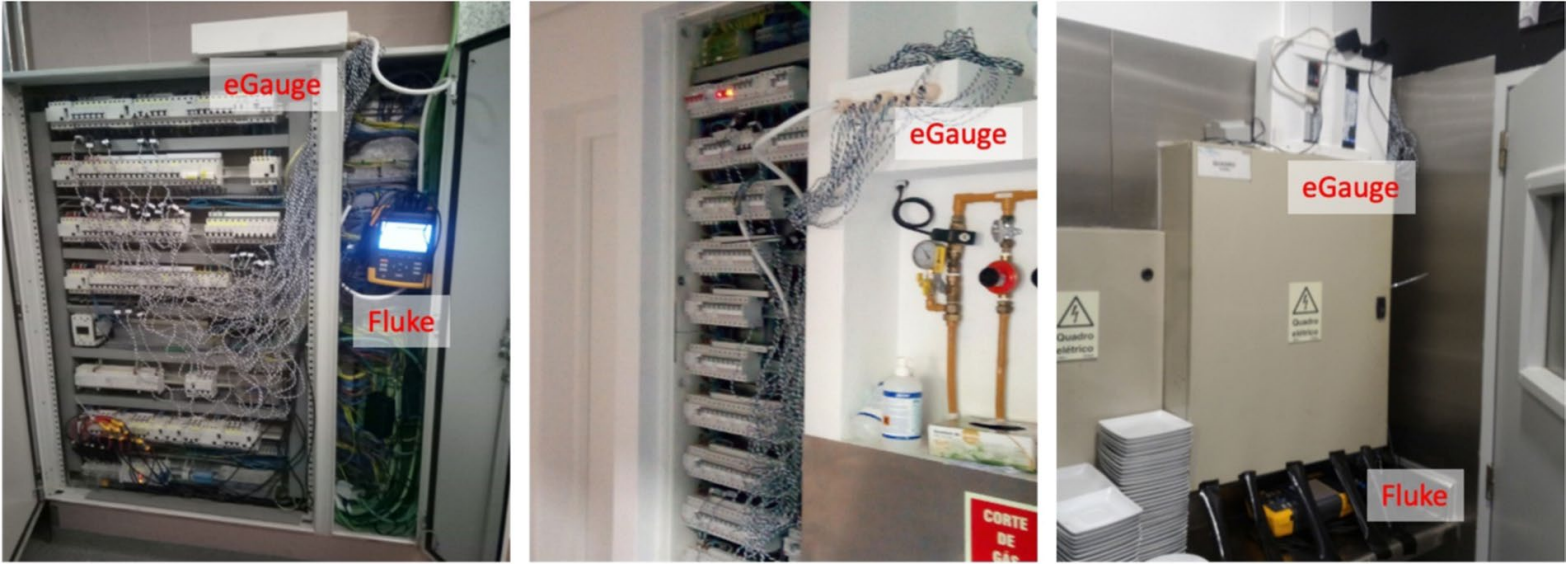
Monitoring system installed in the three IKs: IK 1 (left), IK 2 (middle), and IK 3 (right). Note that the Fluke system was also installed in IK 2, even though it is not shown in the photo.

The monitoring platform was deployed in three restaurant kitchens for consecutive periods between three (IK 2) and four weeks (IK 1 and IK 3). Figure 2 depicts the monitoring systems installed in the main breaker box of each of the three IKs. Table 1 summarizes the details of each IK.

**Table 1:** Summary of the three deployments.

ID	Service	Area (m^2^)	Capacity (Seats)	Start	End	Appliances
IK 1	Dinner	58.15	50	2019-02-06	2019-03-04	18
IK 2	Dinner	25.52	50	2019-03-11	2019-04-03	15
IK 3	Breakfast and Dinner	35.23	40	2019-04-16	2019-05-15	12

A total of 45 appliances were monitored between the three IKs. From these, 27 are single-phase, and 18 are three-phase. A list of the appliances monitored in each IK is provided in Table 2.

**Table 2:** List of the appliances monitored in each industrial kitchen.

Appliance	Phases	IK 1	IK 2	IK 3	Total
Air Conditioner	Three	—	—	1	1
Blast Chiller	Single	1	—	—	1
	Three	—	1	—	1
Cooking Pot	Single	—	1	—	1
Convection Oven	Three	2	1	1	4
Dish Heater	Single	—	—	1	1
Dish Washer	Three	1	—	—	1
Dual Fryer	Three	1	1	—	2
Exhaust	Three	—	1	—	1
Freezer	Single	1	—	2	3
Garde Manger	Single	2	—	—	2
Glass Washer	Single	1	—	—	1
Grill	Three	—	—	1	1
Heating Lamp	Single	—	2	1	3
Hot House	Three	—	1	—	1
Hot Plate Stove	Three	—	—	1	1
Ice Machine	Single	1	—	—	1
Induction Plate	Three	—	1	1	2
Infrared Shelf	Single	1	—	—	1
Microwave	Single	—	1	—	1
Miscellaneous1	Single	—	—	1	1
Mise en Place	Single	1	—	—	1
Refrigerator	Single	4	4	—	5
Refrigerated Showcase	Single	—	—	1	1
Salamander	Three	2	—	1	3
Sous Vide	Single	—	1	—	1
Total	—	18	15	12	45

^1^Blast Chiller; Infrared Lamps; Microwave.

## Data Records

The FIKElecricity dataset is made available individually for each monitored kitchen, and all the data files are in csv format. The data is available on the Open Science Framework (OSF) data repository at 10.17605/OSF.IO/K3G8N^34^.

**Fig. 3:**
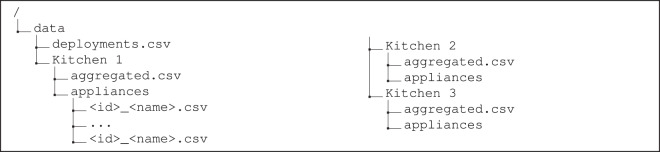
Underlying folder and file organization of FIKElectricity Dataset.

Figure 3 shows an overview of the underlying organization of the FIKElectricity dataset. The following subsections describe the contents of the different files.

### Aggregated consumption measurements

The aggregated consumption measurements are available for each kitchen in a single csv file. The files named aggregated.csv are available in the root folder of each kitchen. The columns of the csv files are described in Table 3. The reported values correspond to the average values within each second.

**Table 3:** Column descriptions in the aggregated consumption files (aggregated.csv).

Column	Description	Units	Column	Description	Units
Timestamp	Timestamp (YYYY-MM-DD hh:mm:ss)	—	S_P1	Apparent Power in Phase 1	*VA*
I_P1	Current RMS in Phase 1	*A*	S_P2	Apparent Power in Phase 2	*VA*
I_P2	Current RMS in Phase 2	*A*	S_P3	Apparent Power in Phase 3	*VA*
I_P3	Current RMS in Phase 3	*A*	PF_P1	Power Factor in Phase 1	—
I_N	Current RMS in Neutral	*A*	PF_P2	Power Factor in Phase 2	—
V_P1	Voltage RMS in Phase 1	*V*	PF_P3	Power Factor in Phase 3	—
V_P2	Voltage RMS in Phase 2	*V*	P_Total	Active Power Total	*W*
V_P3	Voltage RMS in Phase 3	*V*	Q_Total	Reactive Power - Total	*VAR*
V_G	Voltage RMS in Ground	*V*	S_Total	Apparent Power - Total	*VA*
P_P1	Active Power in Phase 1	*W*	PF_Total	Power Factor - Total	—
P_P2	Active Power in Phase 2	*W*	Vn	Voltage Unbalance - Negative Sequence	%
P_P3	Active Power in Phase 3	*W*	Vz	Voltage Unbalance - Zero Sequence	%
Q_P1	Reactive Power in Phase 1	*VAR*	An	Current Unbalance - Negative Sequence	%
Q_P2	Reactive Power in Phase 2	*VAR*	Az	Current Unbalance - Zero Sequence	%
Q_P3	Reactive Power in Phase 3	*VAR*	F	Average System Frequency	*Hz*

### Disaggregated consumption measurements

The files with data for individual appliance consumption are available in the “appliances” folder. For each appliance, there is a csv file, named using the <id>_<name>.csv convention, where <id> refers to the unique identifier of the appliance, and <name> is the appliance name. The underlying fields of the individual appliance consumption files are described in Table 4. The reported current, voltage, and active power values correspond to the meter readings at the given timestamp. The remaining quantities were calculated from the former as per Eqs. (1–7).

**Table 4:** Column description in the appliance consumption files (<id>_<name>.csv).

Column	Description	Units	Column	Description	Units
Timestamp	Timestamp (YYYY-MM-DD hh:mm:ss)	—	Q_P3	Reactive Power in Phase 3	*VAR*
I_P1	Current RMS in Phase 1	*A*	S_P1	Apparent Power in Phase 1	*VA*
I_P2	Current RMS in Phase 2	*A*	S_P1	Apparent Power in Phase 2	*VA*
I_P3	Current RMS in Phase 3	*A*	S_P1	Apparent Power in Phase 3	*VA*
V_P1	Voltage RMS in Phase 1	*V*	PF_P1	Power Factor in Phase 1	—
V_P2	Voltage RMS in Phase 2	*V*	PF_P2	Power Factor in Phase 2	—
V_P3	Voltage RMS in Phase 3	*V*	PF_P3	Power Factor in Phase 3	—
P_P1	Active Power in Phase 1	*W*	P_Total	Active Power Total	*W*
P_P2	Active Power in Phase 2	*W*	P_Total	Active Power Total	*W*
P_P3	Active Power in Phase 3	*W*	Q_Total	Reactive Power - Total	*VAR*
Q_P1	Reactive Power in Phase 1	*VAR*	S_Total	Apparent Power - Total	*VA*
Q_P2	Reactive Power in Phase 2	*VAR*	PF_Total	Power Factor - Total	—

## Technical Validation

### Aggregated consumption

**Fig. 4:**
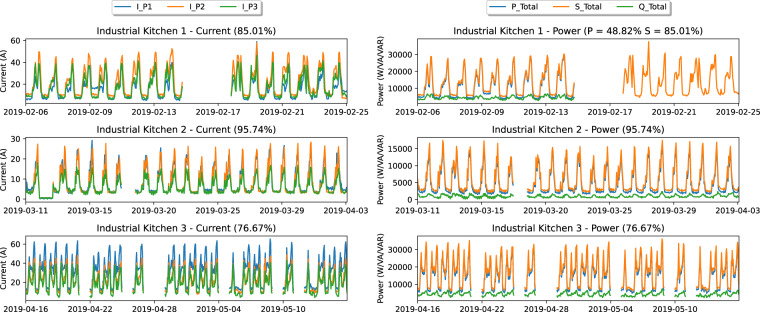
Graphical representations of part of the aggregated consumption measurements.

To illustrate the aggregated consumption data, Fig. 4 depicts some of the measurements contained in the aggregated consumption files. More precisely, the current (left) and power values (right).

First and foremost, it is important to remark that due to a technical issue, the aggregated consumption from IK 1 is only complete until the 15th of February 2019. After that day, only the current, voltage, and frequency measurements were retrieved from the Fluke meter. Regarding IK 2, except for a period between the 2nd and 3rd of March, all the measurements are available (96% of the expected data was collected). As for IK 3, due to technical issues with the installation, there are several periods where the Fluke meter was not collecting data. These periods amount to 24% of the expected data.

### Individual appliance consumption

**Fig. 5:**
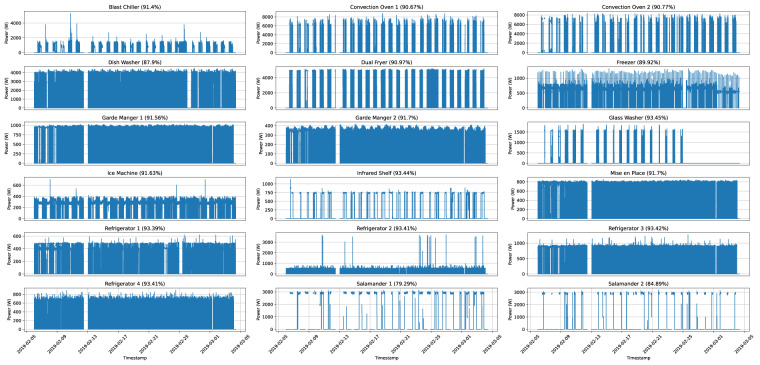
Graphical representation of the measurements obtained for each appliance for the entire deployment duration in IK 1. The data is resampled to $\frac{1}{5}$ Hz.

**Fig. 6:**
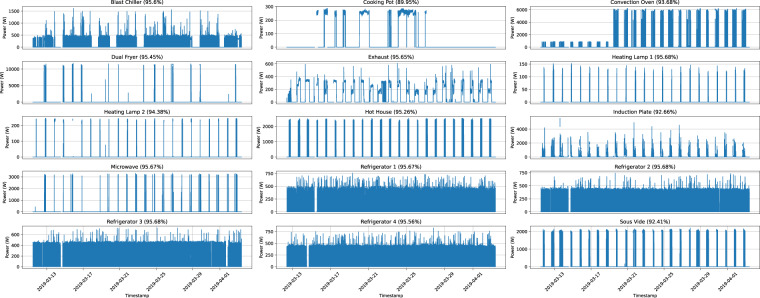
Graphical representation of the measurements obtained for each appliance for the entire deployment duration in IK 2. The data is resampled to $\frac{1}{5}$ Hz.

**Fig. 7:**
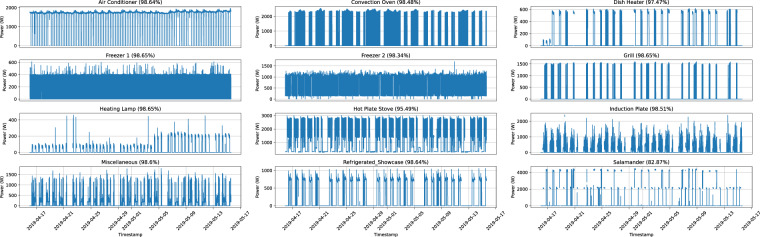
Graphical representation of the measurements obtained for each appliance for the entire deployment duration in IK 3. The data is resampled to $\frac{1}{5}$ Hz.

To illustrate the data collected for each appliance, Figs. 5–7 depicts the measurements obtained for the entire duration of the deployment, resampled to $\frac{1}{5}$ Hz, which was the original rate of acquisition as mentioned in the methods section.

Overall, there are very few gaps in the data. In IK 1, the average percentage of collected samples is 90.74% (standard deviation: 3.5%, minimum: 79%, maximum: 93.45%). For IK 2, the average percentage of complete data is 94.83% (standard deviation: 1.5%, minimum: 89.97%, maximum: 95.68%). Finally, in IK 3, the average percentage is 96.97% (standard deviation: 4.22%, minimum: 82.6%, maximum: 98.63%).

**Fig. 8:**
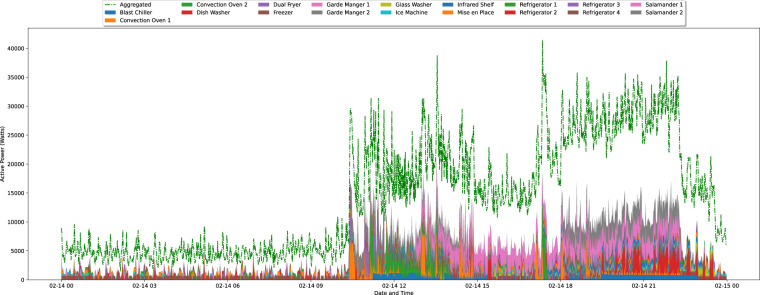
Graphical representation of 24 hours of aggregated and individual appliances consumption from IK 1. The data is resampled to 1/60 Hz.

**Fig. 9:**
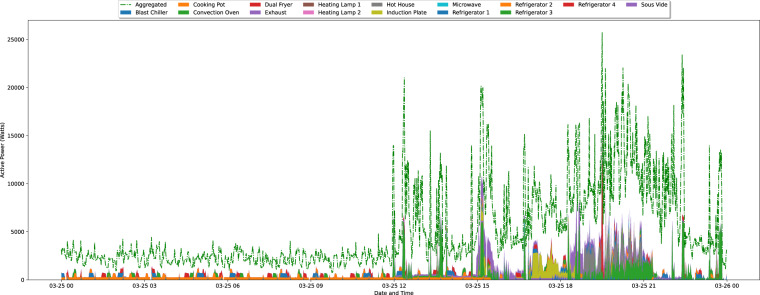
Graphical representation of 24 hours of aggregated and individual appliances consumption from IK 2. The data is resampled to 1/60 Hz.

**Fig. 10:**
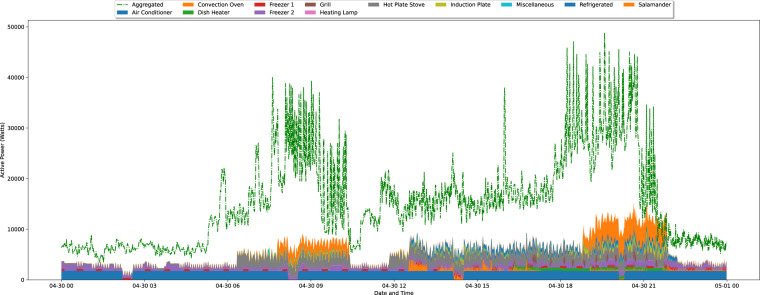
Graphical representation of 24 hours of aggregated and individual appliances consumption from IK 3. The data is resampled to 1/60 Hz.

To further illustrate the collected ground-truth data, Figs. 8–10, depicts one day of aggregated consumption vs. consumption of the individual appliances resampled to 1/60 Hz in each of the three industrial kitchens. In all three graphs, the operation patterns are visible, with IK 1 and IK 2 operating mostly during the afternoon and evening (dinner services), whereas IK 3 operates in the morning and afternoon periods (breakfast and dinner services).

Through these graphs, it is also possible to see that the amount of explained aggregated energy (i.e., the ratio between the consumption of the individual appliances and the aggregated) is considerably low in all the IKs. In this regard, IK 3 has the highest percentage of explained energy (38% on average), despite having fewer individual appliances monitored. There are a few possible explanations for that, including the fact that this was the only IK where the Air Conditioning was monitored. Another reason for the lower explanation percentage of the other IKs is that the aggregated consumption does not include only the kitchen appliances. For example, in IK 1, the aggregated date also includes the demand for a support bar and the dining area. The total demand (in kWh) and percentages of explained energy in the three IKs are summarized in Table 5.

**Table 5:** Total demand for aggregated and individual appliances (in kWh) and the percentage of explained energy in the three IKs.

ID	Day in Figure			Dataset		
	Agg.	App.	Explained (%)	Agg.	App.	Explained (%)
IK 1	337.9	99.7	29.5 (SD: 4.5, Min: 20.8, Max: 38.6)	2561.7	650.1	25.4 (SD: 4.9, Min: 14.9, Max: 30.3))
IK 2	121.7	28.0	23.0 (SD: 5.1, Min: 11.9, Max: 31.2)	2557.5	546.7	22.1 (SD: 3.4, Min: 9.7, Max: 23.2))
IK 3	367.9	121.6	33.0 (SD: 10.7, Min: 20.5, Max: 58.4)	8579.1	3262.5	38.0 (SD: 3.0, Min: 22.9, Max: 39.7))

Agg: Aggregated demand, App: Individual appliances demand.

## Usage Notes

The FIKElectricity dataset is made available in csv format, which is usable in most scientific computing packages, e.g., Python (pandas/numpy), MATLAB (readmatrix), and R(read.csv). Multiple examples of data handling using Python3 can be found in the provided source code.

Concerning the missing data, we deliberately did not clean or strip any of the data. This allows us to retain the data as closely as possible from its raw form. Furthermore, this leaves room for the dataset users to apply their preferred data-cleaning and filling methods. For example, in the case of aggregated demand, the missing data points can be replaced with the sum of all the individual appliances complemented with data generated from a probabilistic distribution of the unexplained demand (i.e., the difference between the aggregated demand and the sum of appliances) estimated from the samples in the vicinity of the missing data points. As for the appliance demand, several methods can be applied depending on the type of appliance and the amount of missing points. Such strategies can vary from quick fixes such as forward-fill or interpolation (linear or weighted) to more advanced techniques using forecasting algorithms, such as the Auto-Regressive Integrated Moving Average (ARIMA)^27^.

This dataset was collected in Madeira Island in Portugal, and during the data collection, two different timezones were observed: WET time until March 31^st^ 2019, and WEST from April 1^st^ 2019. However, to simplify the dataset handling, it was decided to ignore the timezone by representing the timestamps in UTC.

## Data Availability

The code used to collect and store the individual consumption data is available at https://github.com/feelab-info/eGaugeDataAcquisition. The code was developed using Python3.6 and deployed on a Linux machine (Raspbian see http://www.raspbian.org/)). The Python3 code to reproduce the examples presented in this paper is available on the dataset repository at 10.17605/OSF.IO/K3G8N^34^.
